# Supplementary material for: Structural basis for membrane microdomain formation by a human Stomatin complex
Source: Nat Commun. 2025 Aug 12;16:7439. doi: 10.1038/s41467-025-62859-8 (PMC12344292; doi:10.1038/s41467-025-62859-8)
Supplement: Supplementary file 1 — Supplementary Information [file 41467_2025_62859_MOESM1_ESM.pdf]

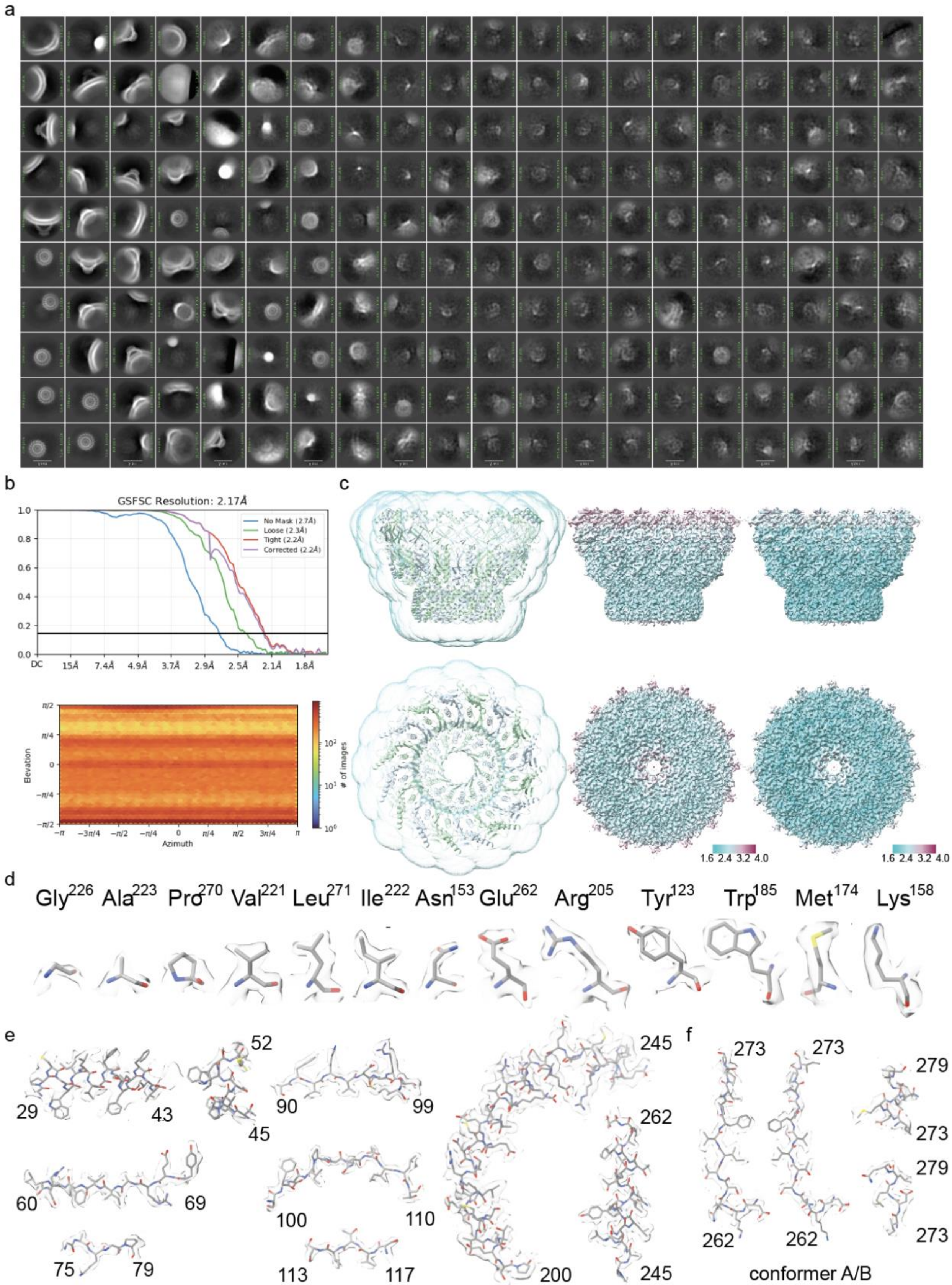
a
b
GSFSC Resolution: 2.17Å
No Mask (2.7Å)
Loose (2.3Å)
Tight (2.2Å)
Corrected (2.2Å)
DC 15Å 7.4Å 4.9Å 3.7Å 2.9Å 2.5Å 2.1Å 1.8Å
Elevation
Azimuth
# of images
c
1.6 2.4 3.2 4.0
d
Gly226 Ala223 Pro270 Val221 Leu271 Ile222 Asn153 Glu262 Arg205 Tyr123 Trp185 Met174 Lys158
e
29 43 52 45 90 99 60 69 100 110 75 79 113 117 245 262 200 245
f
273 273 279 273 279 273 262 262
conformer A/B

Supplementary Fig. 1| Cryo-EM structure of the human Stomatin complex reveals a 16-mer oligomer with alternating conformers.

a, Initial 2D class averages of Stomatin particles show a consistent, closed cap-like morphology. Particles were collected from native vesicles prepared by overexpressing N-terminal ALFA-tagged human Stomatin in HEK293 cells. No incomplete cages were observed.

b, Top: Gold-standard Fourier shell correlation (GSFSC) curves indicate a global resolution of 2.2 Å, with curves shown for unmasked, loosely masked, tightly masked, and corrected maps. Bottom: Angular distribution plot shows uniform sampling of particle orientations across all views.

c, Left: Local resolution estimation mask overlaid with the atomic model of the 16-subunit Stomatin ring, shown in side (top) and top-down (bottom) views. Subunits alternate between Conformer A and Conformer B, depicted in distinct colors. Middle and right: Local resolution maps colored by Fourier shell correlation (FSC) thresholds of 0.5 and 0.143, respectively, with estimated resolution ranging from 1.6 to 4.0 Å.

d, Representative cryo-EM densities with atomic models for selected residues demonstrate high-resolution features and side-chain clarity at 2.2 Å.

e, Atomic model snapshots from key structural regions, including hydrophobic helices (H1, H2), SPFH domains, wall helix, and cap helix.

f, Structural comparison of the β-strands forming the central β-barrel and C-terminal regions of Conformer A and Conformer B. Conformer A terminates in a short helix, while Conformer B ends in a loop. Residue numbers indicate the start and end points of the displayed regions.

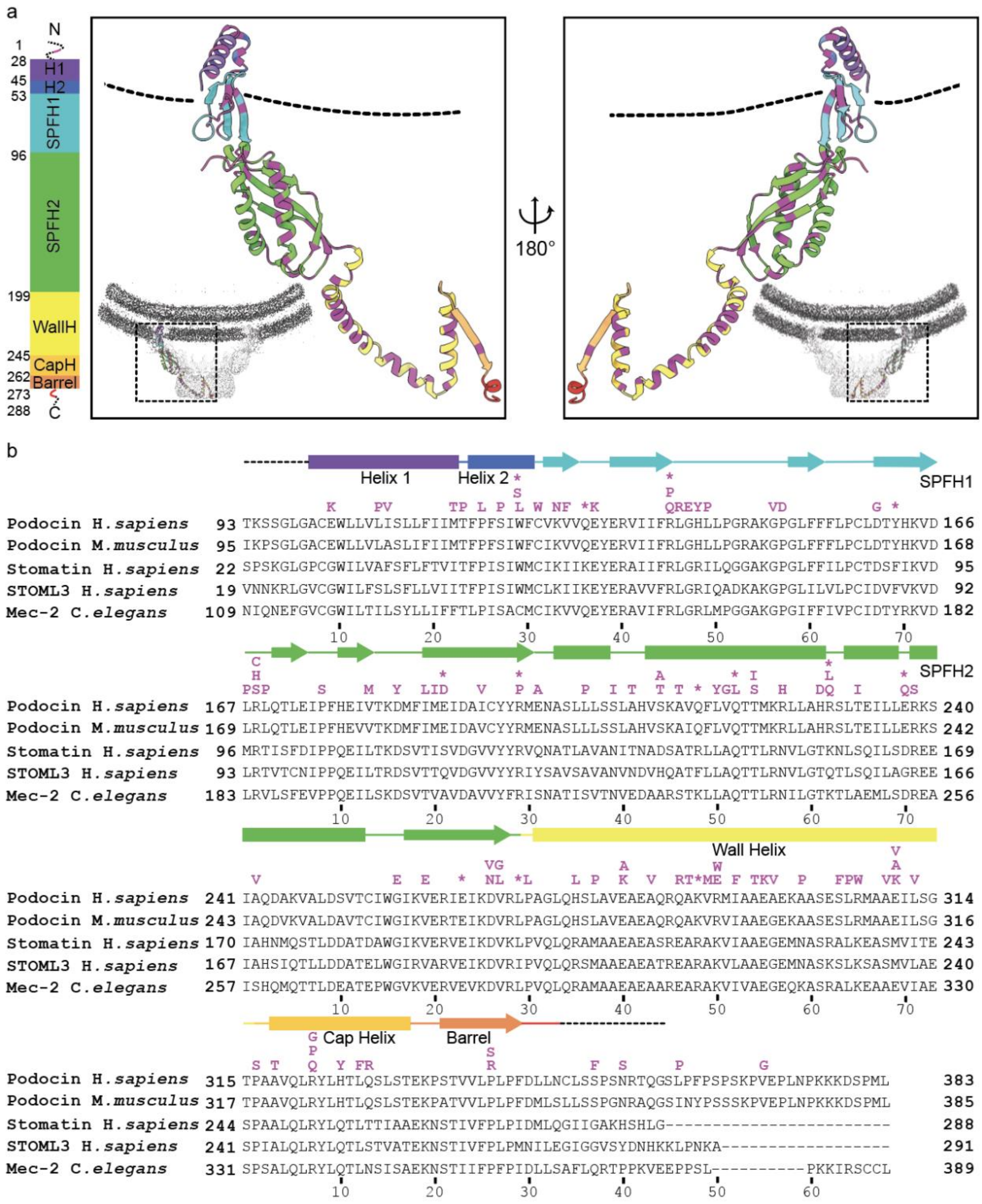

Supplementary Fig. 2| Conserved Membrane-Anchoring Motifs and Disease-Linked Residues in the Stomatin Protein Family a, Side-view images of a single Stomatin protomer highlighting its domain architecture from N- to C-terminus, color-coded as follows: Helix 1 (purple), Helix 2 (blue), SPFH1 (cyan), SPFH2 (green), Wall helix (yellow), Cap helix (orange), β-barrel (orange-red), and C-terminal tail (red). Disease-associated residues are highlighted in

magenta. The dashed black line denotes the approximate position of the membrane. Inset: Cross-sectional density slice showing the orientation of the protomer within the complex and how the N-terminal helices embed into, but do not span, the membrane.

b, Sequence alignment of human Podocin, mouse Podocin, human Stomatin, human STOML3, and *C. elegans* Mec-2, emphasizing domain conservation and secondary structure. Colored bars above the sequences correspond to the structural elements shown in panel a. Disease-linked residues in Podocin are marked in magenta. Key membrane-interacting residues in Stomatin—including C30, C53, C87, and W51—and their Podocin equivalents (e.g., C124, W122) are indicated. Salt-bridge-forming residues (e.g., R67 and D89 in Stomatin; R138 and D160 in Podocin) and other disease-associated sites (e.g., K126) are also annotated. Together, these features support a conserved mechanism of membrane anchoring across Stomatin proteins that is functionally significant and clinically relevant.

Supplementary Table 1. Model Validation.

==

Composition (#)	
Chains	18
Atoms	67450 (Hydrogens: 34780)
Residues	Protein: 4096 Nucleotide: 0
Water	942
Ligands	NA: 16
Bonds (RMSD)	
Length (Å) (# > 4σ)	0.002 (0)
Angles (°) (# > 4σ)	0.492 (0)
MolProbity score	0.60
Clash score	0.28
Ramachandran plot (%)	
Outliers	0.00
Allowed	0.82
Favored	99.18
Rama-Z (Ramachandran plot Z-score, RMSD)	
whole (N = 4032)	1.24 (0.13)
helix (N = 1976)	1.63 (0.12)
sheet (N = 416)	-0.43 (0.22)
loop (N = 1640)	0.30 (0.15)
Rotamer outliers (%)	0.00
Cβ outliers (%)	0.00
Peptide plane (%)	
Cis proline/general	0.0/0.0
Twisted proline/general	0.0/0.0
CaBLAM outliers (%)	0.00
ADP (B-factors)	
Iso/Aniso (#)	32670/0
min/max/mean	
Protein	49.66/100.00/95.33
Nucleotide	---
Ligand	50.00/50.00/50.00
Water	50.00/50.00/50.00
Occupancy	
Mean	1.00

occ = 1 (%)	100.00
0 < occ < 1 (%)	0.00
occ > 1 (%)	0.00

Data

===

Box		
Lengths (Å)	173.02, 172.18, 120.69	
Angles (°)	90.00, 90.00, 90.00	
Supplied Resolution (Å)	2.2	
Resolution Estimates (Å)	Masked	Unmasked
d FSC (half maps; 0.143)	---	---
d 99 (full/half1/half2)	2.3/---/---	2.3/---/---
d model	2.3	2.4
d FSC model (0/0.143/0.5)	2.1/2.3/3.0	2.2/2.4/3.0
Map min/max/mean	-0.36/0.59/0.00	

Model vs. Data

===

CC (mask)	0.59
CC (box)	0.41
CC (peaks)	0.32
CC (volume)	0.59
Mean CC for ligands	0.43
